# Mating Enhances Expression of Hormonal and Trophic Factors in the Midbrain of Female Rats

**DOI:** 10.3389/fnbeh.2020.00021

**Published:** 2020-04-15

**Authors:** Cheryl A. Frye, Sridar V. Chittur

**Affiliations:** ^1^Department of Psychology, The University at Albany-SUNY, Albany, NY, United States; ^2^Department of Biological Sciences, The University at Albany-SUNY, Albany, NY, United States; ^3^Center for Neuroscience Research, The University at Albany-SUNY, Albany, NY, United States; ^4^Center for Life Sciences Research, The University at Albany-SUNY, Albany, NY, United States; ^5^Center for Functional Genomics, The University at Albany-SUNY, Albany, NY, United States

**Keywords:** calbindin, follicle-stimulating hormone, growth hormone, kallikrekin peptidase, luteinizing hormone, thyroid-stimulating hormone

## Abstract

Among female rats, mating enhances neurosteroid formation in the midbrain ventral tegmental area (VTA; independent of peripheral steroid-secreting glands, ovaries, and adrenals). The sources/targets for these actions are not well understood. In Experiment 1, proestrous rats engaged in a mating paradigm, or did not, and the midbrains had been assessed *via* the Affymetrix rat genome microarrays. In Experiment 2, the influence of gonadal and adrenal glands on the expression of these genes was assessed in rats that were proestrous, ovariectomized (OVX), or OVX and adrenalectomized (ADX). The microarrays revealed 53 target genes that were significantly up-regulated (>2.0-fold change) in response to mating. Mating significantly enhanced the midbrain mRNA expression of genes involved in hormonal and trophic actions: Gh1, S100g, and Klk1b3 in proestrous, but not OVX and/or ADX, rats; Fshb in all but OVX/ADX rats; and lutenizing hormone β and thyroid-stimulating hormone (TSH) β in all rats. Thus, mating enhances midbrain gene expression independent and dependent of peripheral glands.

## Introduction

In rats, copulatory behavior is complex, and the manner in which mating occurs can affect the reproductive outcomes. Mating in rats involves a series of mounts, intromissions, and ejaculations (Beach and Jordan, 1956). A mount refers to the male rat positioning his two front paws on the back of the female rat, without penile penetration (Gilbert et al., 1983). Intromissions refer to a slight penetration of the penis into the vagina, which lasts approximately 0.25 s (Peirce and Nuttall, 1961a,b). An ejaculation refers to a deeper intromission, during which the male deposits a sperm plug in the vaginal canal of the female (Matthews and Adler, 1977). A series of mounts and intromissions that ends in ejaculation is called an ejaculatory series. Typically, laboratory rats need 10–20 intromissions before they attain their first ejaculation (Beach and Jordan, 1956).

These standard measures of copulatory behavior are all defined in terms of male behaviors: mounting, intromissions, and ejaculations. However, the rate, timing, and position of all mating behaviors are a result of the interactions between the male and the female of any species (Beach, 1976). Female rats exhibit a species-specific mating behavior. A female rat orients her body toward the male rat as he approaches; she often does this in a hopping manner and then darts away. This type of solicitation, or proceptive behavior, was first described by McClintock and Adler (1978) in a study of a semi-natural environment. The female rat was sometimes observed to put a great deal of space between herself and the male. In a semi-natural environment, the time between intromissions seem to be controlled by female solicitations of males (McClintock et al., 1982). When the size of the cage or a chance for escape allows the female rats to control her interaction with the male rats, her time away is a function dependent upon the preceding behavior(s) of the male (Erskine, 1985). Her time away (i.e., latency period) is the greatest after ejaculations and the shortest after mounts, but in between for intromissions. The percentage of exits is also a result of an antecedent male behavior in that the female will try to get away a fewer times when she receives a mount, as opposed to when she received an ejaculation or intromission (Erskine, 1985).

In 1982, Erskine and Baum found that not only is inter-intromission time dependent upon the preceding male behavior but also the interintromission periods are longer when the female can control the interaction. This phenomenon of female rats being able to escape a male rat by some kind of divided chamber has been termed “pacing” behavior (Gilman and Hitt, 1978). With paced mating, fewer intromissions are necessary to bring about the neuroendocrine response of luteal functioning (Gilman and Hitt, 1978), which results in progesterone secretion, the *sine qua non* for pregnancy. Indeed, paced mating is rewarding for females as it induces a conditioned place preference (Paredes and Alonso, 1997).

Near three scores ago, for my undergraduate thesis, I received a research undergraduate award from NSF to work with Dr. Mary Erskine, a pioneer in the field. I examined the effects of paced or non-paced mating and the time of day of mating in the morning (7–9 am) or in the evening (4–6 pm) to ascertain effects on measures of fertility (number of days in luteal function/pseudopregnancy/pregnancy) and fecundity. Female rats that paced their contacts and mated in the evening were much more likely to become pregnant and have the most pups (Frye and Erskine, 1990; Frye et al., 1998). I went on to show that these effects were due to progesterone’s actions in the midbrain ventral tegmental area (VTA), where there is transient expression of non-estrogen-induced progestin receptors perinatally that remit shortly after birth (Frye, 2001; Blaustein, 2003). Notably, this region is largely devoid of intracellular progestin receptors in adults (Frye, 2001; Blaustein, 2003), but GABA_A_ and NMDARs, co-localized to calbindin-expressing dopaminergic neurons, are prominent (Willick and Kokkinidis, 1995; Westerink et al., 1996; Olson and Nestler, 2007). In hamsters, mating alters gene expression in the diencephalon (i.e., striatum and nucleus accumbens; Bradley et al., 2005).

Progestogens, including progesterone (P) and its metabolites, are pleiotropic factors that can have diverse actions to influence development and/or behavioral processes throughout life. Progesterone and its 5α-reduced metabolite, dihydroprogesterone (DHP), can be secreted from peripheral sources, such as the gonads and the adrenals, to travel through circulation and passively diffuse into target cells in the periphery or brain. The endocrine actions of P or DHP can occur *via* binding to cognate, intracellular steroid receptors (Shughrue et al., 1997) *via* “traditional” actions that modulate gene transcription and translation to protein (Pfaff et al., 1976) in a process that can take approximately 5–10 min to days. These traditional actions of progestogens for reproduction and maintaining pregnancy are well known (Boling and Blandau, 1939; Robson, 1940; Hall, 1956; Erskine, 1989). Moreover, progestogen formation throughout the lifespan may confer protection from neurodegeneration or later central insults (Schumacher et al., 2004; De Nicola et al., 2009; Paris et al., 2011; Garay et al., 2012). Thus, progestogens are important neurotrophic factors that can be naturally enhanced *via* engagement in reproductive behavior.

Some of P’s trophic and behavioral effects may be due to the actions of its neuroactive metabolite that can have rapid actions occurring at “non-traditional” receptor sites (such as membrane-relegated neurotransmitter targets). Progesterone’s 5α-reduced metabolite, DHP, can be further metabolized by 3α-hydroxysteroid dehydrogenase to form 5α-pregnan-3α-ol-20-one (3α, 5α-THP, a.k.a., allopregnanolone). Neurosteroids, such as 3α, 5α-THP, can be synthesized *de novo* in astrocytic and/or neural cells, even in the absence of peripheral steroid sources (gonads and/or adrenals; Baulieu, 1980; Paul and Purdy, 1992; Mellon, 1994). Unlike P and DHP, 3α, 5α-THP is a potent allosteric modulator of GABA_A_ receptors (Majewska et al., 1986; Callachan et al., 1987; Fodor et al., 2005), where it can promote rapid (<10 min) effects (Baulieu, 1980; Gee et al., 1995; Frye and Vongher, 1999). 3α, 5α-THP may also allosterically modulate glutamatergic N-methyl-D-aspartate receptors (NMDARs; Frye and Paris, 2011) and has less well-defined actions through other non-steroidal, ligand-gated, ion channel, and/or G protein-coupled receptors (Rupprecht and Holsboer, 1999). Thus, P can have rapid, non-traditional actions in the brain *via* conversion to its metabolite, 3α, 5α-THP.

In rodents, mating is utilized as a bioassay to determine the mechanisms that underlie the steroids’ effects. Female rodents are dependent upon the central actions of P, and/or its metabolites, to promote lordosis (a stereotypical posture that allows mating to occur). In hamsters, mating alters gene expression in the diencephalon (i.e., striatum and nucleus accumbens; Bradley et al., 2005). We have utilized a paced mating paradigm, wherein female rats are given free access to a chamber containing a confined male and an empty chamber. In this paradigm, females can temporally pace their mating contacts (Erskine, 1985). Engaging in this type of mating enhances 3α, 5α-THP content *via* neurosteroidogenesis in the midbrain, hippocampus, frontal cortex, and diencephalon (Frye and Rhodes, 2006; Frye et al., 2007). Moreover, these effects have been localized to the midbrain VTA, an important region for motivated behavior and reward (Bain and Kornetsky, 1989; Agars and Kokkinidis, 1992; Frye et al., 1992; McBride et al., 1993). Notably, this region is largely devoid of estradiol-induced intracellular progestin receptors (Frye, 2001; Blaustein, 2003), but GABA_A_ and NMDARs, co-localized to calbindin-expressing dopaminergic neurons, are prominent (Willick and Kokkinidis, 1995; Westerink et al., 1996; Olson and Nestler, 2007). Blocking 3α, 5α-THP formation, or these neurotransmitter receptors and their downstream signal transduction targets, within the VTA attenuates lordosis (Frye et al., 2008a; Frye and Paris, 2011; Frye, 2011). Thus, engagement in paced mating is an important stimulator of neurosteroidogenesis in the midbrain VTA among female rats, and mating-induced enhancement of 3α, 5α-THP in the VTA is critical for the maintenance of rodent reproduction.

The mechanisms that may underlie mating behavior *via* non-traditional actions in the midbrain remain to be elucidated. We hypothesized that engagement in paced mating would alter the gene expression of traditional, and/or non-traditional, steroid targets in the midbrain, the latter of which may include GABAergic, dopaminergic, and glutamatergic substrates and/or their downstream signal transduction processes. Further, we hypothesized that gonadal and/or adrenal steroid production might play an important role in the expression of these and/or other important trophic factors: there are rapid changes in the midbrain VTA in response to progestogens, and mating can rapidly increase progestogens (Frye, 2011). As such, RNA microarrays were performed on sexually receptive (proestrous) female rats that engaged in paced mating with a male or proestrous rats that were exposed to an empty, clean paced mating chamber in the absence of a male. To ascertain the roles of peripheral progestogen sources, some targets were followed up with quantitative polymerase chain reaction (qPCR) in midbrain tissues from proestrous rats that were gonadally intact, had their ovaries removed (OVX), or had their ovaries and adrenals (OVX/ADX) removed.

## Materials and Methods

These methods were approved by the Institutional Animal Care and Use Committee at The University at Albany-SUNY and were conducted in accordance with the ethical guidelines defined by the National Institutes of Health (NIH Publication No. 85-23).

### Study Procedure

Rats that were gonadally intact and had regular 4- to 5-days estrous cycles (*n* = 6) underwent paced mating for one ejaculatory sequence (*n* = 3) or were yoked controls that were exposed only to a clean paced mating chamber (*n* = 3). The rats were euthanized immediately following behavioral testing as described below. Frozen midbrain tissue was assessed *via* RNA microarrays, as described below, at the Center for Functional Genomics, The University at Albany. RNA expression differences in some targets that were of interest were verified *via* qPCR. Some changes that occur in midbrain gene expression with mating may be due to the actions of progestogens; however, given that neural cells can synthesize progestogens in the absence of peripheral production (King, 2008) and may serve as important trophic factors, it was important to include extirpation groups that had minimal circulatory progestogens. In order to ascertain the degree to which peripheral progestogen secretion influenced changes in RNA expression, some rats were OVX (*n* = 6) or were OVX/ADX (*n* = 6), estradiol-benzoate (EB)-primed (10 μg, SC), and tested 10 days after surgery. Therefore, the influence of peripheral progestogens on central changes in gene expression that occurred with mating among extirpation groups is assumed to be limited. Ovariectomized or OVX/ADX rats were primed with EB (10 μg, SC) and tested 44 h later in the paced mating task or as yoked controls. This EB priming regimen has been utilized to facilitate mating and produce physiological estradiol and P in OVX or OVX/ADX rats previously (Frye et al., 2008b; Frye and Paris, 2011) and is necessary so that comparisons could be completed in rats that engaged in mating, which otherwise would not occur without EB priming. Midbrain was collected from rats that were OVX and/or ADX, and RNA was isolated in these tissues as described. qPCR was run in these tissues to assess the ovarian and/or adrenal contributions to up-regulation of targets delineated in the microarray experiments.

### Animals and Housing

Adult (50–60 days old) Long–Evans female rats (*N* = 25) were bred in the Life Sciences Laboratory Animal Care Facility at The University at Albany-SUNY (original stock obtained from Taconic Farms, Germantown, NY, USA). The rats were housed in polycarbonate cages (45 × 24 × 21 cm) with woodchip bedding in a temperature-controlled room (21 ± 1°C) and were maintained on a 12:12-h reversed light cycle (lights off at 08:00 h) with continuous access to Purina rat chow and tap water in their home cages.

### Determination of Estrous Cycle Phase in Gonadally Intact Rats

Vaginal epithelium was collected from gonadally intact rats (*n* = 13) for 21–22 consecutive days by inserting an eye dropper with distilled water into the vaginal canal and squeezing. Epithelial samples were assessed under a light microscope to determine the phase of the estrous cycle as per previously described methods (Long and Evans, 1922; Frye et al., 2000; Marcondes et al., 2002). Briefly, samples characterized by numerous epithelial cells that were nucleated were considered to be of the proestrous phase; the time when circulatory E levels are elevated, progestogen levels are rising, and female rats are sexually receptive. The samples that were characterized by the presence of many cornified cells were considered to be of the estrous phase. A lack of nucleated or cornified cells, combined with the presence of leukocytes, was indicative of the diestrous phase. The presence of all three cell types, in similar proportions, was considered to be indicative of the meta-estrous phase. Six of the 13 gonadally intact rats in the present study had typical 4- to 5-days estrous cycles across the 21- to 22-days period and were utilized for behavioral testing.

### Surgical Protocol for Ovariectomized and/or Adrenalectomized Rats

The rats underwent surgery using xylazine (12 mg/kg) and ketamine (70 mg/kg) anesthesia. Ovariectomy was performed on some rats (*n* = 12) as previously described (Frye et al., 2008b). Briefly, a ~3-cm incision was made in the dorsal region of the flank, just anterior to the kidney. The ovary was isolated from the surrounding adipose tissue, ligated with surgical silk (4–0 USP, 1.5 m), and extirpated. The muscle wall and skin were closed with 2–3 silk sutures, and the procedure was repeated for the ovary on the alternate side. Some rats also underwent ADX (*n* = 6) at the time of OVX as per prior methods (Rhodes et al., 2004). For ADX, the adrenal gland was located *via* proximity to the kidney; the gland was isolated with a forceps and extirpated. One OVX/ADX rat died prior to surgical completion. Following surgery and prior to testing, the animals were monitored for changes in weight, righting response, flank stimulation response, and/or muscle tone (Marshall and Teitelbaum, 1974). No rats failed these assessments in the present study. Given that ADX rats are rendered sodium deficient, they were provided continuous access to a bottle of sodium chloride (0.9%) in addition to drinking water. All rats that had surgery were allowed 10 days for recovery and hormonal washout prior to testing.

#### Behavioral Outcome Measures

Paced mating was conducted as per previous methods (Erskine, 1985). In brief, the paced mating apparatus (37.5 × 75 × 30 cm) was equally divided by a Plexiglass partition, which contained a small (5 cm in diameter) hole in the bottom center, allowing the female (but not the stimulus male) free access to both sides of the apparatus. The frequency of mating contacts (intromissions) was recorded, and a lordosis quotient was calculated [(frequency of female dorsiflexion during a sexual contact/total sexual contacts by a male) * 100] during a 15-min test. The frequency of proceptive/solicitation behavior (ear wiggling, hopping, and darting) prior to intromission was recorded and calculated as a proceptivity quotient [(frequency of proceptive behaviors/total sexual contacts by a male) * 100]. The percentage of defensive aggressive behaviors (vocalizing and attack) that females displayed in response to male intromission was calculated as a defensive aggression quotient [(frequency of defensive aggression/total sexual contacts by a male) * 100]. Following intromission, the percentage of times the experimental female left the chamber containing the male (% exits) following sexual contacts was recorded. The effects of ovarian and/or adrenal gland extirpation to reduce the sexual response of female rodents to male mounting have been established (Davidson et al., 1968; Komisaruk and Diakow, 1973; Feder et al., 1974), and the present behavioral data is confirmatory in this regard.

### Tissue Collection and Dissection

Immediately following the behavioral testing, the rats were euthanized *via* rapid decapitation in an RNase/DNase-free environment. All surfaces, tools, and personal protective equipment that were utilized for euthanasia were decontaminated with quatricide and RNase/DNase cleansing solution (RNase AWAY, Laboratory Products Sales, Rochester, NY, USA) prior to termination of each subject. For each subject, whole brains were removed at the time of death, and the brain was positioned ventral side up for gross dissection of the midbrain immediately after the brain was extracted from the skull. The optic chiasm was utilized as the anterior border of the dissection, the pontine regions were utilized as the posterior border, and the cerebral aqueduct was utilized as the ventral border. The borders were ~1.5 mm from the anterior, posterior, and midline as previously reported (Frye et al., 2007). Dissected midbrains were placed in RNase/DNase-free tubes and immediately flash-frozen on dry ice and maintained at −80°C until microarray.

### Microarray Analyses

Tissues from gonadally intact rats (*N* = 6; chamber-exposed, *n* = 3, paced mated, *n* = 3) were sent to the microarray core at the Center for Functional Genomics at the University at Albany-SUNY to have the RNA extracted. Then, they were sent along to UCLA Microarray Core Facilities to the consortium coordinators, Brandy Hamill and Stanley Nelson, to complete the Affymetrix Rat Genome 230 2.0 gene chip as described below. See the following files from the UCLA Microarray Consortium for the raw data of Experiment 1 comparing the intact paced mated and the non-mated rats (frye-affy-rat-483660). The effects on mating of the midbrain gene expression of OVX and OVX/ADX hormone-primed rats (Experiment 2) are on file at UCLA Microarray Consortium files frye-affy-rat-584452 and 584783, respectively. All of the data are available at the following website: https://www.ncbi.nlm.nih.gov/geo/query/acc.cgi?acc=GSE127272.

In brief, tissue preparation was performed as described in the Affymetrix GeneChip Expression Analysis Manual (Affymetrix, Santa Clara, CA, USA). RNA was isolated from tissue using an RNeasy RNA Isolation kit (Qiagen, Valencia, CA, USA), which included an on-column Dnase step. The integrity of isolated RNA was assessed by using a nanodrop spectrophotometer and an Agilent Bioanalyzer. RIN between 8.5 and 10 for all samples and RNA between 50 and 200 μg/sample were detected. RNA was converted to single-stranded cDNA using Superscript II reverse transcriptase and the GeneChip T7 promoter primer kit (Affymetrix, Santa Clara, CA, USA). The single-stranded cDNA was converted to double-stranded cDNA using DNA polymerase I, DNA ligase, and RNase H from *Escherichia coli*. Double-stranded cDNA was cleaned up and *in vitro*-transcribed to biotin-labeled cRNA. There were 35–200 base fragments generated by metal-induced hydrolysis and hybridized to Affymetrix Rat Genome 230 2.0 oligonucleotide arrays. After hybridization, the chip was washed and stained with streptavidin–phycoerythrin before being scanned. An antibody amplification staining protocol that uses biotinylated goat IgG, followed by a second streptavidin–phycoerythrin staining, increases the sensitivity of the assay. The chip was then scanned, and images were analyzed qualitatively using the Affymetrix GeneChip Operating System software. Further analyses of the data were done in GeneSpring v7.3, wherein the data were normalized using GC robust multi-array average (GCRMA). The signals were also baseline-transformed to the median of all samples, following which the probe sets were filtered to exclude those with signal values less than the 20th percentile across all conditions. This list was subjected to an ANOVA (*p* < 0.05) with a Benjamini–Hochberg false discovery rate correction to identify statistical differences between all conditions vs. control. The statistically significant genes were subjected to a twofold change filter to identify genes that were differentially expressed in paced vs. in non-paced animals. The *raw data* from our microarray analyses is accessible at (Accession: GSE127272) a public data repository through the UCLA Microarray Consortium reference files (frye-affy-rat-483660, 584783, and 584452) for intact, OVX, and OVX/ADX rats, respectively.

### Quantitative Real-Time Polymerase Chain Reaction

Some genes that were revealed to be significantly different in paced and non-mated rats in Experiment 1 were followed up with qPCR in gonadally intact, OVX, and OVX/ADX tissues. In order to avoid the inclusion of tissues from another sample, the same tissues that were utilized for the microarray were utilized for qPCR analyses, yielding *n* = 3/condition. RNA was isolated and converted to cDNA as described above. qPCR assays were run using the iTaq SYBR green supermix with ROX (Bio-Rad, Hercules, CA, USA) on a 7900HT platform (Applied Biosystems). The final reaction volume was 20 μl, comprised of iTaq SYBR green master mix (10 μl), nuclease-free dH_2_O (4.96 μl), forward and reverse primers (0.02 μl each), and 20 ng cDNA template (5 μl). Nuclease-free dH_2_O was utilized in place of cDNA for a non-template control (NTC). Reactions were run in triplicate. Each plate had represented a negative (NTC) and positive control (β-actin or Gapdh reference gene). These normalizing control/housekeeping genes were selected because their expression did not change per microarray data. Reactions were incubated for 10 min at 95°C to activate iTaq polymerase, followed by 40 cycles consisting of 15 s at 95°C and elongation at 60°C for 10 s. After each cycle, fluorescence was measured.

#### Primers

The primers were designed using the Primer Express software (Applied Biosystems, Foster City, CA, USA) and were validated *via* BLAT for specificity and selectivity. All primers were synthesized by Eurofins MWG Operon (Huntsville, AL, USA) and were reconstituted to a 100-μM concentration. The forward and reverse primers are indicated in Table 1.

**Table 1:** mRNA sequences that were used forward and backward (1st and 2nd lines) and were investigated with quantitative real-time polymerase chain reaction (qPCR).

GenBank Accession #	Gene Symbol	mRNA sequences first line forward/second line backward
NM_022384	Ascl1	5′-GGGGGCGGTCACAAGTCAGC-3′
		5′-ACTTGACCCGGTTGCGCTCG-3′
NM_053918	Cga	5′-CCCACTCCCGCCAGGTCCAA-3′
		5′-TCAGCAGTCGTCAGCGCAGC-3′
D00577	Fshb_1_	5′-GCCCGCCACTCAGACTCCCT-3′
		5′-GCCCTGGCACTCCCAGTCCT-3′
M36804	Fshb_2_	5′-AGATTGCCTGGCTGTGCCGC-3′
		5′-GCCCCAGTGCAGAGCAGACC-3′
NM_012858	Lhb	5′-TACAAAGAGTTCGAGCGTGCC-3′
		5′-AATGGAATAGCGCTGTCCCTC-3′
NM_031038	Gnrhr	5′-ATCGCTCCCTGGCCGTCACT-3′
		5′-AAACTGCTGGCCCAGAGCCG-3′
NM_057187	Grifin	5′-AAGGTGCTCCAGTTCCCACAT-3′
		5′-TCTGGTGATGGTAGCTAGCGGT-3′
NM_031523	Klk1b3	5′-GCTGCTCACTGCGCAACCGA-3′
		5′-CCGAGGCAAGGCAGGTGCTC-3′
NM_012858	Lhb	5′-GAATGGAGAGGCTCCAGGGGCT-3′
		5′-GAATGGAGAGGCTCCAGGGGCT-3′
NM_012629	Prl	5′-CAGCCAAGTGTCAGCCCGGA-3′
		5′-GTGTCTGGCAGTCGCCACCA-3′
AI230625	S100g	5′-TGGCAGCACTCACTGACAGCA-3′
		5′-TGGACAGCTGGTTTGGATCGCC-3′
M10902	Tshb	5′-CACCACCATCTGCGCTGGGT-3′
		5′-TTGCAGCTCAGGGCAACGGG-3′

### Statistical Analyses

Independent student’s *t*-tests were used to assess differences of extirpation condition between groups (gonadally intact, OVX, and OVX/ADX) on behavioral endpoints as compared to intact conditions. For each qPCR analyte, the results were calculated with the Applied Biosystem’s Sequence Detection Software and calculated *via* the ΔΔCt method, with the mean of control rats utilized as the calibrator for each experimental rat. The qPCR results were analyzed by student’s *t*-tests on extirpation (gonadally intact, OVX, and OVX/ADX) and mating condition (chamber-exposed and paced-mated) to assess between-groups differences. All data are expressed as mean ± SEM. The effects were considered as significant when *p* < 0.05.

## Results

### Behavioral Endpoints

**Figure 1:**
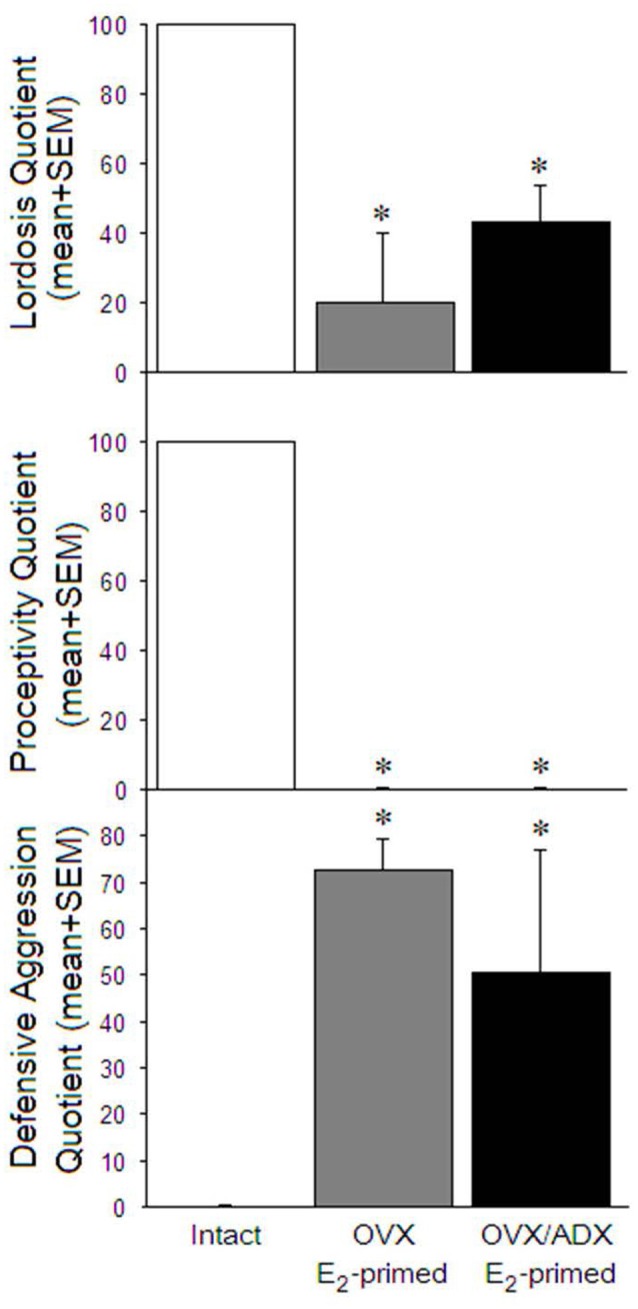
Lordosis quotients (top) and proceptivity quotients (middle) were significantly higher, and defensive aggression quotients (bottom) significantly lower, among naturally receptive rats (left), compared to ovariectomized estradiol primed (middle) and ovariectomized and adrenalectomized estradiol primed (right) sexually-naïve male rats. GH1 was elevated most among naturally mated rats, and was increased similarly among estradiol primed rats whether mated or not. *p < 0.05.

Extirpation of the ovaries and/or adrenals significantly reduced engagement in lordosis (*t_OVX_*_(7)_ = 6.11, *p* < 0.05; *t_OVX/ADX_*_(7)_ = 8.17, *p* < 0.05; Figure 1, top), reduced proceptive solicitations (*t_OVX_*_(7)_ = 10.64, *p* < 0.05; *t_OVX/ADX_*_(7)_ = 10.64, *p* < 0.05; Figure 1, middle), and increased defensive aggression (*t_OVX_*_(7)_ = −14.08, *p* < 0.05; *t_OVX/ADX_*_(7)_ = −2.84, *p* < 0.05; Figure 1, bottom) in response to sexual contact by a male compared to gonadally intact proestrous rats.

### Microarray Endpoints

Differences in RNA expression were detected among several genes in gonadally intact, proestrous rats. There were 53 genes that had greater than 2-fold enhancement of RNA expression among paced mated rats compared to chamber-exposed controls (Table 3).

**Table 2:** mRNA that were significantly (>2-fold change) up-regulated in the midbrain among rats that were paced mated compared to yoked control rats that were only exposed to the paced mating chamber (*n* = 3/group).

Up-regulated Genes			
GenBank Accession #	Gene Symbol	Gene Name	Fold Change in Paced Mated vs. Chamber-Exposed
V01238	Gh1	growth hormone 1	162.6
NM_012629	Prl	prolactin	58.7
NM_012521	S100g	S100 calcium binding protein G	32.0
D00577	Fshb_1_	follicle-stimulating hormone, β polypeptide	21.4
M36804	Fshb_2_	follicle-stimulating hormone, β polypeptide	16.1
NM_012858	Lhb	luteinizing hormone β	10.3
NM_031038	Gnrhr	gonadotropin releasing hormone receptor	9.0
NM_031523	Klk1b3	kallikrein 1-related peptidase b3	8.3
NM_022384	Ascl1	achaete-scute complex homolog 1 (Drosophila)	7.3
NM_053918	Cga	glycoprotein hormones, α polypeptide	6.0
M10902	Tshb	thyroid stimulating hormone, β	4.6
NM_057187	Grifin	galectin-related inter-fiber protein	2.9
BG669096	Mpz	myelin protein zero	9.2
NM_133563	Giot1	gonadotropin inducible ovarian transcription factor 1	6.5
NM_017027	Mpz	myelin protein zero	5.2
AF260741	Gpha2	glycoprotein hormone α 2	5.1
NM_053572	Pcdh21	protocadherin 21	4.7
NM_024388	Nr4a1	nuclear receptor subfamily 4, group A, member 1	4.5
NM_031796	Galnt5	UDP-N-acetyl-α-D- galactosamine:polypeptide N-acetylgalactosaminyltransferase 5 (GalNAc-T5)	3.5
AF200684	Slc7a7	solute carrier family 7 (cationic amino acid transporter, y+ system), member 7	3.4
NM_022526	Dap	death-associated protein	3.4
AI230625	LOC6848 71	similar to Protein C8orf4 (Thyroid cancer protein 1; TC-1)	3.2
NM_031972	Aldh3a1	aldehyde dehydrogenase 3 family, member A1	3.2
NM_012999	Pcsk6	proprotein convertase subtilisin/kexin type 6	3.0
NM_031808	Capn6	calpain 6	2.8
AA819329	RGD130 5347	similar to RIKEN cDNA 2610528J11	2.8
AF214568	Enpep	glutamyl aminopeptidase	2.7
NM_019237	Pcolce	procollagen C-endopeptidase enhancer	2.7
AA819629	Ifi44l	interferon-induced protein 44-like	2.6
BI278379	Rcn3	reticulocalbin 3, EF-hand calcium binding domain	2.6
NM_053744	Dlk1	Delta-like 1 homolog (*Drosophila*)	2.6
NM_053750	Nppc	natriuretic peptide precursor C	2.6
NM_052805	Chrna3	cholinergic receptor, nicotinic, α 3	2.5
M83681	Rab3d	RAB3D, member RAS oncogene family	2.5
NM_013069	Cd74	Cd74 molecule, major histocompatibility complex, class II invariant chain	2.5
AI029410	Fndc3c1	fibronectin type III domain containing 3C1	2.4
NM_053819	Timp1	tissue inhibitor of metalloproteinase 1	2.4
Y00480	RT1-Da	RT1 class II, locus Da	2.4
AF065147	Cd44	Cd44 molecule	2.4
NM_031334	Cdh1	cadherin 1	2.4
NM_021663	Nucb2	nucleobindin 2	2.3
NM_033237	Gal	galanin prepropeptide	2.2
NM_057194	Plscr1	phospholipid scramblase 1	2.2
NM_031817	Omd	Osteomodulin	2.2
NM_019296	Cdk1	Cyclin-dependent kinase 1	2.1
NM_012760	Plagl1	pleiomorphic adenoma gene-like 1	2.1
NM_031511	Igf2	insulin-like growth factor 2	2.1
L07646	Gnrhr	gonadotropin releasing hormone receptor	2.1
NM_019354	Ucp2	uncoupling protein 2 (mitochondrial, proton carrier)	2.1
M23995	Aldh1a7	aldehyde dehydrogenase family 1, subfamily A7	2.0
NM_012949	Eno3	enolase 3, β, muscle	2.0
AF419342	Sytl4	synaptotagmin-like 4	2.0
NM_022232	Dnajc3	DnaJ (Hsp40) homolog, subfamily C, member 3	2.0

*Those highlighted in gray were followed up on with qPCR (see Table 3)*.

**Table 3:** qPCR results for nine genes that were significantly (>2-fold change) up-regulated in the midbrain of rats that were paced mated compared to yoked control rats that were only exposed to the paced mating chamber (*n* = 3/group).

Relative expression differences *via* qPCR (mean ± SEM) vs. mean of control rat expression						
	Chamber-exposed			Paced mated		
Gene symbol	Gonadally intact	OVX	OVX/ADX	Gonadally intact	OVX	OVX/ADX
Prl	373.5 ± 373.0	0.8 ± 0.4	0.3 ± 0.3	1276.7 ± 1018.9	1.5 ± 0.3	5.0 ± 2.8
Fshb_1_	19.4 ± 18.8	0.7 ± 0.6	0.3 ± 0.3	150.2 ± 95.1	3.6 ± 2.4	19.7 ± 19.3
Fshb_2_	0.8 ± 0.4^∧^	0.8 ± 0.4^∧^	0.3 ± 0.3	2.1 ± 0.7^∧^	1.5 ± 0.3^∧^	0.2 ± 0.2
Lhb	1.3 ± 0.8	1.2 ± 0.7	0.3 ± 0.3	10.1 ± 6.5*	8.4 ± 5.7*	6.2 ± 3.1*
Gnrhr	2.6 ± 2.1	0.8 ± 0.4	0.3 ± 0.3	34.0 ± 19.4	1.5 ± 0.3	3.4 ± 3.1
Ascl1	1.0 ± 0.2	0.8 ± 0.4	0.3 ± 0.3	5.5 ± 2.8	1.5 ± 0.3	0.2 ± 0.2
Cga	3.1 ± 1.9	2.3 ± 1.6	0.3 ± 0.3	2.1 ± 0.7	1.9 ± 0.9	0.1 ± 0.1
Tshb	0.4 ± 0.3	0.5 ± 0.3	0.3 ± 0.3	1.4 ± 0.2*	2.2 ± 0.9*	0.4 ± 0.4*
Grifin	3.9 ± 1.7	4.4 ± 2.9	2.0 ± 1.5	10.8 ± 3.0	2.4 ± 0.7	4.2 ± 3.6

Probes were conducted in gonadally intact rats, ovariectomized (OVX) rats, and OVX/adrenalectomized (OVX/ADX) rats that were chamber-exposed or paced mated. *Indicates a main effect for paced mated rats significantly differing from chamber-exposed control rats; ^∧^Indicates a main effect for groups significantly differing from OVX/ADX rats, *p* < 0.05.

### qPCR Among Gonadally Intact, OVX, and/or ADX Rats

**Figure 2:**
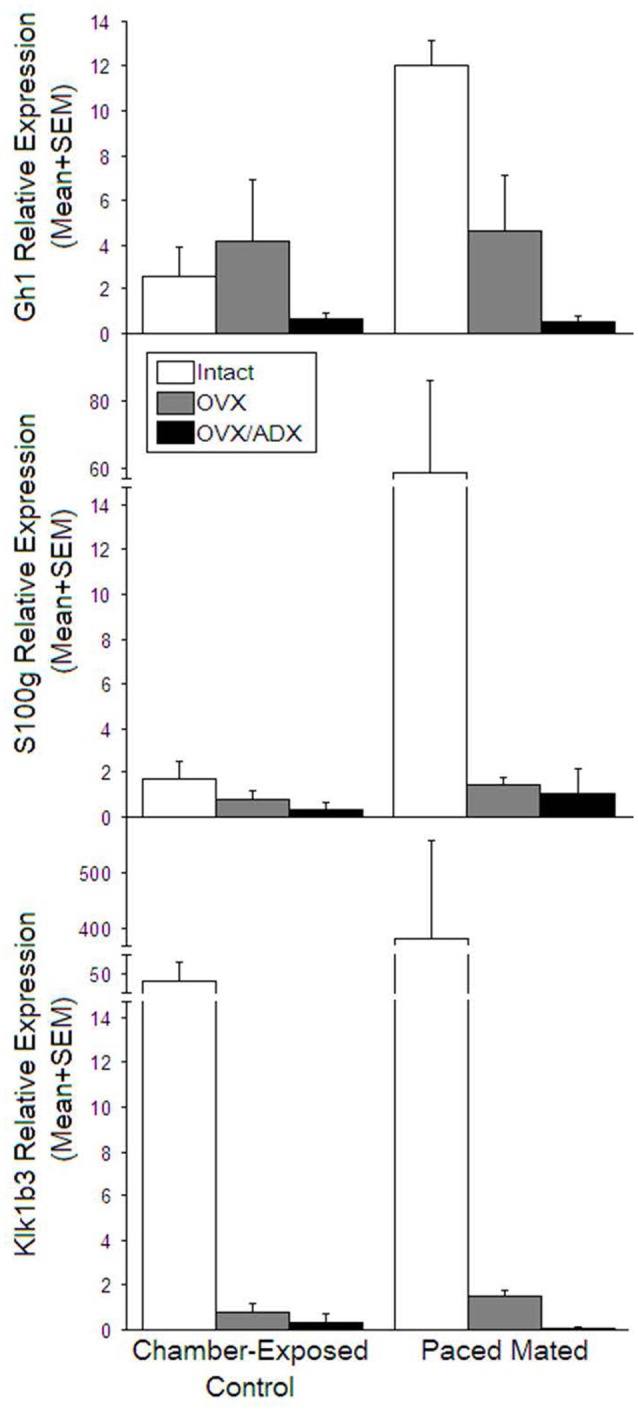
Relative expression among among naturally receptive rats (open bars), ovariectomized estradiol primed (gray bars) and ovariectomized and adrenalectomized estradiol primed (black) that were just exposed to the mating chamber left side of graph or were actually mated (right side of graph). Top panel: GH1 was elevated most among naturally mated rats, and was increased similarly among estradiol primed rats whether mated or not. Middle panel S100 expression was increased most among naturally mated rats. Bottom panel kallikrekin 1-related peptidase b3 (Klk1b3, a.k.a.Ngfg) was increased in naturally receptive rats irrespective of mating.

Of the genes that were up-regulated, the most robust enhancement was observed in growth hormone 1 (Gh1). The extirpation status significantly influenced the mRNA expression, such that Gh1 expression was greater among gonadally intact (*t*_(10)_ = 2.99, *p* < 0.05) or OVX (*t*_(10)_ = 2.26, *p* < 0.05) rats compared to OVX/ADX rats (Figure 2, top). Similarly, kallikrekin 1-related peptidase b3 (Klk1b3, a.k.a. Ngfg) was significantly greater among gonadally intact rats compared to OVX (*t*_(10)_ = 1.87, *p* < 0.05) or OVX/ADX rats (*t*_(10)_ = 1.88, *p* < 0.05; Figure 2, bottom). The extirpation condition significantly influenced the expression of the follicle-stimulating hormone β polypeptide (Fshb). The expression of Fshb was greater among gonadally intact (*t*_(10)_ = 1.14, *p* < 0.05 or *t*_(10)_ = 0.72, *p* < 0.05) rats compared to OVX/ADX rats (Table 2).

Engagement in paced mating was also observed to significantly enhance two genes irrespective of peripheral gland extirpation. The expression of lutenizing hormone β (Lhb; *t*_(16)_ = −2.71, *p* < 0.05) and thyroid-stimulating hormone β (Tshb; *t*_(16)_ = −2.28, *p* < 0.05) were significantly enhanced among mated, compared to chamber-exposed, rats (Table 2). Notably, the latter effect was not due to an enhancement in Tshb among the mated OVX/ADX rats (Table 2). Fshb expression did not significantly differ among the mated rats compared to the chamber-exposed rats.

Gonadotropin-releasing hormone receptor and achaete-scute complex homolog 1 did not significantly differ among groups (Table 2). Other genes that were probed for validation, prolactin, glycoprotein hormone α polypeptide, and galectin-related inter-fiber protein, did not significantly differ between extirpation and mating groups (Table 2).

## Discussion

Our hypothesis that engagement in paced mating would alter the gene expression of steroid targets in the midbrain, including those that may be involved in GABAergic, dopaminergic, and glutamatergic signaling and/or their downstream signal transduction processes, was upheld. Further, among rats that engaged in paced mating, the mRNA of 53 genes was significantly up-regulated in the midbrain compared to those of the chamber-exposed controls. Engaging in paced mating enhances neurosteroidogenesis in the midbrain (Frye and Rhodes, 2006; Frye et al., 2007). As such, GABAergic, dopaminergic, and glutamatergic signaling may be influenced by these changes in gene expression, albeit it must be noted that this represents only a few aspects of the functions that are mediated by the genes that were up-regulated. The present results support and extend those of previous studies to suggest some of the hormonal factors for these effects of paced mating. Our hypothesis that mating can enhance trophic factors was upheld. An unexpected finding was that the most highly up-regulated genes in the midbrain (by microarray analysis) were pituitary hormone genes [alpha glycoprotein, TSHb, LHb, FSHb, growth hormone (GH), and prolactin (PRL)] and GnRH. There was no downregulation of gene expression with mating.

### Gh1

Paced mating may enhance GH expression in the midbrain, and these actions may contribute to progestogens’ effects to maintain mating. In the present investigation, an interaction was observed, wherein Gh1 was enhanced by paced mating among gonadally intact rats, but not among OVX or OVX/ADX rats. Moreover, ADX rats had the lowest expression of Gh1 compared to the other groups. It is notable that 3α, 5α-THP content is co-expressed with GnRH (which promotes GH secretion) *in vitro* (Buchanan et al., 2000), 3α, 5α-THP promotes GnRH secretion (El-Etr et al., 1995; Sim et al., 2001), and infusion of GnRH facilitates lordosis of E-primed OVX rats *via* inhibition of VTA neurons (similar to 3α, 5α-THP; Sirinathsinghji et al., 1995; Suga et al., 1997). Lesioning dopamine neurons in the VTA *via* infusion of 6-hydroxydopamine (which preferentially depletes dopaminergic and noradrenergic neurons) exacerbates this effect (Sirinathsinghji et al., 1995), suggesting that the actions of GnRH neurons in this region may present a separate, but necessary, component mediating the maintenance of lordosis within the midbrain. In particular, in the midbrain central gray, β-endorphin has been demonstrated to inhibit lordosis, but this effect is overcome by the prior infusion of GnRH (Sirinathsinghji et al., 1995), supporting the notion that tonic inhibition of GnRH neurons in this region is upstream of opioid effects on lordosis. As such, the enhancement of midbrain Gh1 mRNA, *via* engagement in paced mating, may play an important role in the feedback from ovarian-derived, and adrenally derived, sex steroids that maintain mating once initiation has occurred.

The enhancement of Gh1 mRNA may also confer some of progestogens’ neuroprotective effects. Gonadal steroids, such as central progestogens and peripheral E, mediate pulsatile GH secretion from pituitary in people and rodent models (Hohmann et al., 1998; Veldhuis, 1998). These effects are typically considered to occur *via* the actions of steroids at target sites within the hypothalamus and pituitary. In particular, E can act at growth hormone-releasing hormone receptor to promote GH and PRL release (Simard et al., 1986; Martel et al., 1990). Growth hormone is trophic and, throughout development, plays an important role in the maintenance of skeletal mass (Harris and Heaney, 1969; Stuart and Lazarus, 1975). Innervation of GnRH neurons *via* neurosteroid targets is enhanced cyclically. Among young adult rats in proestrous (the high-progestogen phase of the estrous cycle), the number of vesicular GABA transporter terminals that synapse on GnRH neurons is decreased and the number of vesicular glutamate transporter-2 terminals is increased compared to those in diestrous (the low progestogen phase of the estrous cycle; Khan et al., 2010). This variation is attenuated among middle-aged rats, suggesting that hormone decline drives these plastic effects (Khan et al., 2010). Thus, Gh1 mRNA enhancement, *via* exposure to paced mating, may promote trophic actions in the brain and may be partly dependent on peripheral ovary and adrenal signaling.

### Lhb, Tshb, and Fshb

Engaging in paced mating may enhance the secretion of gonadotropin β subunits independent of peripheral steroidogenesis. Irrespective of whether the animals were intact, OVX, or OVX/ADX, Lhb and Tshb were significantly up-regulated in the midbrain with mating. Others have demonstrated that 3α, 5α-THP dose-dependently enhances luteinizing hormone and follicle-stimulating hormone secretion in OVX E-primed rats, effects that could be attenuated by pretreatment with a GABA_A_, but not progestin receptor, antagonist (Murphy and Mahesh, 1984; Brann et al., 1990). While Lhb is typically considered with respect to its pulsatile secretion from the anterior pituitary to stimulate P production from ovaries (Dalkin et al., 2001; Ascoli et al., 2002), these data demonstrate Lhb mRNA enhancement in a gonad-independent manner in the midbrain (albeit this effect was greatest among gonadally intact rats). While steroid-independent examples of gonadotropin-modulating genes exist (Matagne et al., 2009), such expression is developmentally regulated. The activational effects of E to negatively regulate Lhb are critical in rodent hypothalamus and are thought to occur *via* classic steroid–DNA interactions (Glidewell-Kenney et al., 2008). However, in the midbrain, LH synthesis in response to mating may be gonad/adrenal independent and/or insufficiently repressed *via* systemic E-priming. Apart from the well-studied actions in the hypothalamus, LH may interact with important effectors at the level of the midbrain. Iontophoresis of LH-releasing hormone (LHRH) in the midbrain central gray enhances the neuronal membrane sensitivity among OVX E-primed rats (Schiess et al., 1987). Noradrenaline is also shown to stimulate LH secretion, in part, *via* actions in the dorsal noradrenergic tracts of the midbrain (Sar and Stumpf, 1981). Stimulating these midbrain tracts reduces LH secretion, supporting the importance of LH modulation at the level of the midbrain (Bergen and Leung, 1988). Similarly, Tshb was enhanced with paced mating irrespective of peripheral gland extirpation. [^3^H]thyroid-stimulating hormone (TSH) accumulates in the midbrain (Bhargava et al., 1989), and noradrenergic projections from the locus coeruleus to the hypothalamus have been suggested to play a lesser role in TSH secretion (Jaffer et al., 1990). The nigrostriatal dopamine system has been demonstrated to modulate TSH secretion (Männistö et al., 1981), and the present data support the notion that engagement in paced mating can facilitate actions at Tshb in the midbrain. Unlike Lhb and Tshb, Fshb was reduced among OVX/ADX rats compared to that in intact or OVX rats. Fshb feedback is also independent of classic E actions in the mouse hypothalamus (Glidewell-Kenney et al., 2008). As such, these data support the notion that E may also play an important role in sensitizing gonadotropin β subunits *via* noradrenergic stimulation in the midbrain, and these actions may be facilitated by engagement in mating to further promote progestogen biosynthesis to maintain mating.

The gonadotropin substrates revealed in the present study likely demonstrate an interaction between the midbrain and the hypothalamic processes. The sequential actions of E and P in the ventromedial hypothalamus are critical for the initiation of lordosis and appear to be upstream of steroids’ actions in the midbrain, which maintain lordosis and mediate the appetitive aspects of mating (solicitations and proceptive behavior; Mong and Pfaff, 2004). Prolactin cell bodies, localized to the arcuate and ventromedial nuclei of the hypothalamus, project to the midbrain central gray (Harlan et al., 1989). Luteinizing hormone releasing hormone, PRL, and TSH excite spontaneous activity of periaqueductal gray neurons in the midbrain (Ogawa et al., 1992). Further, administration of GABA_A_ agonists modulates these effects (Ogawa et al., 1992). Thus, gonadotropins in the midbrain and the hypothalamus act as important mediators of central signaling and influence the effects of peripherally, and centrally, derived sex steroids.

### S100g and Klk1b3

The enhanced mRNA of neurotrophic factors with paced mating may confer some of progestogens’ neuroprotective effects. Engaging in paced mating enhances the expression of S100g mRNA in the midbrain among intact, but not OVX or OVX/ADX rats. S100g (calbindin D28k) is a calcium-binding protein that is best studied for its presence in non-degenerating midbrain dopaminergic neurons in Parkinson’s disease (Lavoie and Parent, 1991; Lavoie et al., 1991; Mouatt-Prigent et al., 1994). In owl monkeys, calbindin-immunoreactive neurons are characterized by noradrenergic innervation (Gaspar et al., 1992). In rats, dopaminergic calbindin-positive cells are expressed in VTA, can be GABAergic, and are resistant to depletion *via* the 6-hydroxydopamine toxin (Sarabi et al., 2001). Similarly, in the present study, Klk1b3 mRNA up-regulation was observed markedly among gonadally intact rats, but not among OVX or OVX/ADX rats. Klk1b3 (Ngfg) protein is a trophic factor and may partly underlie the neuroprotective effects of progestogens in the brain. In rat models of traumatic brain injury or ischemic insult, the administration of P, or 3α, 5α-THP, reduces edema and tissue degeneration following the insult and improves the morphological and functional outcomes (He et al., 2004; Schumacher et al., 2004; Sayeed et al., 2007). Progesterone treatment reduces the mRNA and the protein markers of apoptosis, such as Bax and Bad in the cerebral cortex of rats that have had traumatic brain injury (Yao et al., 2005). Thus, the present data showing changes in S100g and Klk1b3 mRNA with paced mating suggest that these factors may be involved in the effects of paced mating to promote neuroprotective effects, partly *via* the neurosteroidogenesis of progestogens, in a peripheral gland-dependent manner.

### Limitations of These Findings

As in all scientific work, there are limitations. Those that have limited our study are as follows: first, we were limited by the number of microarray analyses provided (*n* = 18). As such, we have small samples, which is typically not a problem in gene-related studies that generates volumes of data. However, this precluded ANOVAs for behavioral studies or attributional statistics about particular aspects of behavior and how it influenced gene expression. Indeed we were fortunate to find a manageable number of factors (*n* = 53), many of which relate to our prior investigation that were identified in the midbrain, as well as some new and interesting targets. Second, among our study’s novel findings was that most of the highly up-regulated genes found were for pituitary glycoprotein hormones. Indeed the most highly up-regulated genes in the midbrain (by microarray analysis) were pituitary hormone genes (alpha glycoprotein, TSHb, LHb, FSHb, GH, and PRL). However, there was no change in GnRH gene expression as detected by microarray (although the receptor did increase slightly). The expression of these genes in the midbrain, while consistent with some data on peripheral expression/secretion, is curious. We discuss how changes in midbrain gene expression among intact and/or those with ovaries and/or adrenals may deplete peripheral progestogen sources to influence these effects, albeit these glands are not purely progestogen secreting. For example, PRL expression is completely ablated by ovariectomy, an effect known to be dependent on estrogens. As such, we cannot rule out the role of estrogens, glucocorticoids, and/or mineralocorticoids in these processes. As discussed above, we propose that the PRL neurons in the hypothalamus project to the midbrain central gray, wherein there are many connections to the VTA below. Alternatively, there could be pituitary secretion of the said hormones in response to lordosis and modulation of midbrain neuronal activity. In order to address this, up-regulated proteins for pituitary glycoprotein hormones could be confirmed in another way, such as immunohistochemistry. However, there are just as many challenges with that technique, and given the number of factors to investigate, it is beyond the scope of the present work but will be an important topic of future investigations.

## Conclusion

Engagement in paced mating is dependent on progestogens’ actions in the midbrain and involves gonadotropin signaling, which may be important for neurosteroidogenesis, maintenance of reproduction, and/or trophic signaling. However, it must be noted that changes in gene expression cannot be solely attributed to the pacing aspects of mating in the present study. The changes observed in gene expression could be due to mating in any condition and are not necessarily associated with the pacing of the mating contacts. Future investigations should aim to assess the effects of non-paced vs. paced mating on gene expression in the brain in order to parse out the influence of consummatory vs. appetitive aspects of mating. Moreover, the direct influence of 3α, 5α-THP in these effects remains implied but unknown. Microarrays of rat midbrain tissue confirmed the presence of the pregnane xenobiotic receptor (PXR; albeit the RNA expression of PXR was not mating dependent). Ongoing work is focused on investigating the role of PXR, which may act upstream of mitochondrial mechanisms to enhance *de novo* 3α, 5α-THP production, and the subsequent effects on mating (Frye, 2011; Frye et al., 2011). Future investigations should aim to assess the influence of intra-VTA PXR and/or other biosynthetic factors upstream of 3α, 5α-THP formation in the midbrain. Despite these uncertainties, the present report demonstrates a mating-enhanced mRNA expression of gonadotropin β subunits (Lhb, Tshb, and Fshb) and neurotrophic factors (Gh1, S100g, and Klk1b3) in the midbrain. These targets may present novel substrates for neurosteroids’ reproductive and/or neuroprotective effects.

The most important results of the present study were the changes in the expression of the midbrain neurosteroid genes in relation to the mating rate of females. Using microarray methods, as many as 53 genes, involved in the expression of neurosteroids, trophic factors, and pituitary hormones, differed in the proestrous paced mating group compared to the OVX/ADX groups who mated less. Thus, data on the importance of mating in relation to the greatest changes among neurosteroid, trophic, and pituitary genes were observed.

## Data Availability Statement

The datasets generated for this study can be found in the UCLA GSE microarray consortium.references files (frye-affy-rat-483660, 584783, 584452) for intact, ovx and ovx adx rats, respectively.

## Ethics Statement

The animal study was reviewed and approved by UAlbany IACUC (Institutional Animal Care and Use Committee).

## Author Contributions

SC assisted Jason Paris with running the microarray samples in his laboratory. CF acquired funding for the project as a supplement to her R01 examining the role of neurosteroids in the midbrain VTA in social, cognitive, and reward behavior and assisted Jason Paris through all phases of the project.

## Conflict of Interest

The authors declare that the research was conducted in the absence of any commercial or financial relationships that could be construed as a potential conflict of interest.
